# Treatment of Transthyretin Amyloid Cardiomyopathy: The Current Options, the Future, and the Challenges

**DOI:** 10.3390/jcm11082148

**Published:** 2022-04-12

**Authors:** Carsten Tschöpe, Ahmed Elsanhoury

**Affiliations:** 1 Berlin Institute of Health (BIH) Center for Regenerative Therapies (BCRT), Campus Virchow Clinic, Charité–University Medicine Berlin, D-13353 Berlin, Germany; ahmed.elsanhoury@charite.de; 2 German Centre for Cardiovascular Research (DZHK), Partner Site Berlin, D-13353 Berlin, Germany; 3 Department of Cardiology, Campus Virchow Klinikum, Charité–University Medicine Berlin, D-13353 Berlin, Germany

**Keywords:** transthyretin amyloid cardiomyopathy, TTR amyloid, cardiac amyloidosis, tafamidis, clinical development

## Abstract

Transthyretin amyloid cardiomyopathy (ATTR-CM) is a progressively debilitating, rare disease associated with high mortality. ATTR-CM occurs when TTR amyloid protein builds up in the myocardium along with different organs, most commonly the peripheral and the autonomic nervous systems. Managing the cardiac complications with standard heart failure medications is difficult due to the challenge to maintain a balance between the high filling pressure associated with restricted ventricular volume and the low cardiac output. To date, tafamidis is the only agent approved for ATTR-CM treatment. Besides, several agents, including green tea, tolcapone, and diflunisal, are used off-label in ATTR-CM patients. Novel therapies using RNA interference also offer clinical promise. Patisiran and inotersen are currently approved for ATTR-polyneuropathy of hereditary origin and are under investigation for ATTR-CM. Monoclonal antibodies in the early development phases carry hope for amyloid deposit clearance. Despite several drug candidates in the clinical development pipeline, the small ATTR-CM patient population raises several challenges. This review describes current and future therapies for ATTR-CM and sheds light on the clinical development hurdles facing them.

## 1. Introduction

Amyloidosis is a rare disease in which amyloid fibrils misfold and aggregate into toxic oligomers that build up extracellularly in the tissue [1]. It can affect different organs, most commonly the heart, kidneys, liver, spleen, peripheral nerves, and gastrointestinal tract [1,2]. There are different types of amyloidosis according to the nature of the misfolded protein. Immunoglobulin light chain amyloidosis (AL) and transthyretin amyloidosis (ATTR) are the main two types of amyloidosis that affect the heart [2,3]. The first results from abnormal plasma cell production of monoclonal light chain fragments that misfold, causing myocardial toxicity [2,4]. The latter results from the dissociation and misfolding of the transthyretin (TTR), the protein that transports thyroxine (T4) and retinol-binding protein (RBP) in the serum and cerebrospinal fluid [2,5]. Both forms together account for nearly 95% of all cardiac amyloidosis cases [2,3,6]. TTR is mainly produced by the liver (90%) and circulates as a homotetramer. The choroid plexus and retinal epithelium produce the remaining 10% [7,8]. Destabilization of the tetramer results in the dissociation and aggregation of amyloid fibrils, which then accumulate in the interstitial space of various organs, causing progressive loss of function [9,10]. Transthyretin amyloid cardiomyopathy (ATTR-CM) is a rare, progressively debilitating disease that occurs when TTR builds up in the myocardium along with the peripheral and autonomic nervous systems [10]. ATTR-CM often presents with progressive heart failure with right- and left-sided symptoms, complicated by arrhythmias, most commonly atrial fibrillation, owing to conduction abnormalities [10,11]. However, cardiac symptoms are often proceeded by musculoskeletal manifestation (e.g., carpal tunnel syndrome) [12]. There are two forms of ATTR-CM, hereditary and wild type. Hereditary ATTR-CM (ATTRv) is characterized by a single amino acid substitution caused by a point mutation in the *TTR* gene, located on the long arm of chromosome 18 [13,14]. The most common mutation in ATTRv-CM is V122I, present in African Americans at a prevalence of 3.4% [15]. The prevalence of the V122I mutation in non-African American descents is low or possibly under-estimated. Wild-type ATTR-CM (ATTRwt), previously termed senile cardiac amyloidosis (SSA), is a non-familial form of the disease that predominantly presents in elderly male patients [16,17]. Both ATTR-CM forms are associated with impaired survival rates. The median survival of untreated ATTR-CM is 2.5 years for ATTRv V122I and 3.6 years for ATTRwt [18]. ATTR-CM is usually misdiagnosed, particularly early in its course owing to non-specific symptoms and multi-system involvement [19,20]. Lately, ATTR-CM is increasingly being recognized in clinical practice as a result of increased disease awareness and enhanced diagnostic techniques. Echocardiography-measured strain indices, advanced magnetic resonance imaging modalities, including T1 mapping and extracellular volume evaluation, as well as bone scintigraphy provide the necessary tools for the non-invasive diagnosis of ATTR-CM [20]. Moreover, genetic testing is instrumental to confirm the diagnosis of ATTRv. A definitive diagnosis could be reached with the aforementioned non-invasive tools, yet endomyocardial biopsy (EMB) evaluation is recommended in cases where AL amyloidosis cannot be ruled out via serum analysis [21]. However, histologic differentiation between ATTR and AL amyloidosis cannot be based solely on the Congo red staining. There is a compelling medical need to develop therapies for ATTR-CM, being an incapacitative illness that is eventually fatal if left untreated [22]. For many decades, the treatment of ATTRv-CM consisted of liver transplantation to remove the source of misfolded TTR or combined liver/heart transplantation, besides symptomatic treatments or solely heart transplantation in advanced ATTRwt-CM [23]. After the positive results of the ATTR-ACT study [24], the United States Food and Drug Administration (FDA) and the European Medicines Agency (EMA) have granted tafamidis 61 mg capsules marketing authorization for ATTRv and ATTRwt-CM. However, so far, no treatment can reverse the disease. This review discusses the current and future therapies for ATTR-CM and the hurdles facing their clinical development.

## 2. Symptomatic Treatments

The distinctive pathophysiology of ATTR-CM, characterized by both restrictive ventricular filling and reduced stroke volume, renders the use of standard heart failure medications difficult [18], summarized in Table 1. Loop diuretics are vital to reduce cardiac and peripheral congestion, especially in patients with right ventricular (RV) congestion and pulmonary edema, which subsequently relieves dyspnea [18,25,26], preferably taken in combination with aldosterone antagonists to prevent hypokalemia. However, blood pressure must be monitored adequately, since reducing preload may adversely affect renal perfusion and cardiac output [18]. Drugs acting on the renin-angiotensin system are poorly tolerated since they exacerbate hypotension, especially in patients with amyloid-associated autonomic dysfunction [18,26]. Beta-blockers are also poorly tolerated due to their negative chronotropic effect, given the restricted ventricular volume and the reliance of the cardiac output on the heart rate [18,26]. Calcium channel blockers and digoxin are generally contraindicated due to their profound binding to amyloid, which intensifies their pharmacologic effects and can result in cardiac rhythm disturbances or sudden death [18,26,27]. However, digoxin can be an option for pulse control in uncontrollable tachyarrhythmia absoluta. Alpha-1-adrenoreceptor agonists such as midodrine can treat orthostatic hypotension allowing higher doses of diuretics [28]. Amiodarone is the anti-arrhythmic agent of choice for patients with atrial fibrillation/flutter [28]. Oral anticoagulants are particularly required for patients with atrial fibrillation since blood stasis is a major source of thromboembolic events [18,29,30]. Pacemaker implantation is necessary for up to 25–36% of ATTRv patients and as many as 43% of ATTRwt patients [26,31]. However, in ATTR-CM patients pacemaker implantation is associated with high risk and worse survival [32]. There is no clear indication for prophylactic implantable cardioverter-defibrillator (ICD) therapy in ATTR-CM, since arrhythmias are not the primary cause of cardiac death in this population, except for patients with a high burden of ventricular tachycardias [33]. In general, the symptomatic management of ATTR-CM follows the CHAD-STOP concept: conduction and rhythm disorders prevention, high heart rate maintenance, anticoagulation, diuretics, and STOP ß-receptor and calcium-channel blockers, digoxin, and renin-angiotensin-aldosterone inhibitors [34].

## 3. Supportive Therapy with Epigallocatechin-3-Gallate (Green Tea)

Epigallocatechin-3-gallate (EGCG) is a polyphenolic natural compound most abundant in green tea [35]. EGCG is well tolerated, without major safety concerns. It has been shown to disaggregate amyloid fibrils and prevent TTR formation [36]. An observational study reported that the treatment of ATTR-CM patients with green tea extract for nine months was able to reduce left ventricular (LV) wall mass and thickness [36]. However, in a single-center retrospective study, patients who received a 675 mg daily dose of EGCG for a minimum of nine months did not gain any survival benefit compared to patients on solely symptomatic treatment [37].

## 4. Specific Treatments

**Figure 1:**
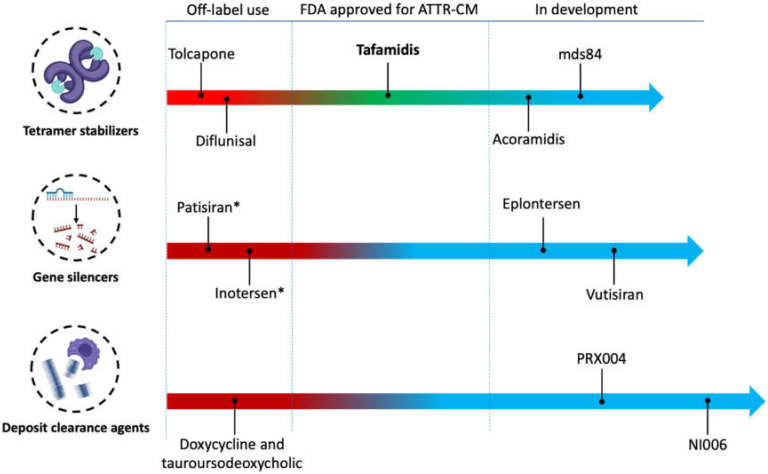
The clinical development pipeline of therapies for transthyretin amyloid cardiomyopathy (ATTR-CM). * Approved by the American food and drug administration and the European medicine agency for the treatment of ATTR-polyneuropathy. Figure cartoons were created with Biorender.com (accessed on 11 March 2022).

For many decades, liver transplantation has been considered the only approach to halt the progression of ATTRv-CM. Although liver transplantation removes the main source of aberrant amyloid protein, it does not provide a complete cure for the disease. In a large collective of 2127 liver transplant patients who were included in Familial Amyloidotic Polyneuropathy World Transplant Registry (FAPWTR), the overall 20-year survival rate was 55.3%. The largest benefit was observed in ATTRv patients carrying the most common pVal50Met variant [38]. The reason behind the incomplete cure is the aggregation of newly formed amyloid around the preexisting amyloid seeds [39]. Combining liver and heart transplantation can overcome this setback [40]. Moreover, this treatment approach is restricted by the availability of transplant organs as well as the advanced age and comorbidities of most ATTR-CM patients, which limits the feasibility of undergoing surgery and the need for life-long immunosuppression therapy [41]. Accordingly, there is a great need for ATTR-CM-specific pharmacological treatments. In 2019, the U.S. Food and Drug Administration (FDA) approved tafamidis at a dose-strength of 61 mg capsule as the first pharmacologic treatment for ATTR-CM of any kind. Different pharmacological treatments are used off-label or in the development pipeline. However, none of them is yet approved for ATTR-CM, as illustrated in Figure 1.

### 4.1. Transthyretin Tetramer Stabilization

Dissociation of the TTR tetramer is considered the rate-limiting step in the amyloidogenesis [42]. The binding of T4 into one of the two interdimeric binding pockets of TTE increases the stability of the tetramer. When both binding pockets are occupied, the tetramer becomes more stable. However, the binding of T4 to one of the pockets exerts a negative cooperativity effect that reduces the accessibility to the second binding pocket by undergoing a conformational change [43].

#### 4.1.1. Tolcapone

Tolcapone is an orally available catechol-O-methyltransferase inhibitor used for the treatment of Parkinson’s disease, usually in combination with levodopa and carbidopa. It has a high binding affinity to the TTR binding pockets, stabilizing the tetramer [44]. Tolcapone is used as an off-label medication for ATTR-CM. The results of a phase IIa study (NCT02191826) support the repurposing of tolcapone for both ATTRwt and ATTRv. The study showed that tolcapone increases TTR stability by 52% 2 h following a single 200 mg dose. Patients who received 100 mg of tolcapone every 4 h showed a 38.8% increase in TTR stability 2 h following the first dose, and this effect was maintained for 24 h [45]. Owing to its high central nervous system (CNS) penetrance, tolcapone was evaluated in a proof-of-concept study as a possible treatment for leptomeningeal ATTR, a rare CNS form of ATTR. The study was completed in April 2019, but the results are not yet published (NCT03591757). Despite these promising results, the clinical application of tolcapone in ATTR-CM is rather difficult. The drug has a short half-life, which mandates short dosing intervals. The side effects of tolcapone are severe, including hepatotoxicity, sleep disturbances, dyskinesia, and gastrointestinal disturbances. In addition, the drug is metabolized through the CYP2C9 enzyme which causes interactions with a wide range of medications, e.g., warfarin and sulfonylurea [46,47,48].

#### 4.1.2. Diflunisal

Diflunisal is an FDA-approved non-steroidal anti-inflammatory agent (NSAID). Due to its structural match to T4, diflunisal can bind and stabilize the TTR tetramer [42]. A randomized placebo-controlled clinical trial showed that diflunisal significantly reduces the neurological impairment and preserved quality of life of familial amyloid polyneuropathy, a neurological phenotype of ATTR [49]. The results of an open-label observational study that monitored the effect of diflunisal 500 mg daily support its efficacy in ATTRv patients. However, around 19% of the patients dropped out due to typical NSAID side-effects of diflunisal, most prominently gastrolesivity, cardiotoxicity, and nephrotoxicity [50]. A retrospective cohort study of 35 patients with ATTRwt-CM showed that diflunisal administration improved survival and overall stability in clinical echocardiographic disease markers [51]. Another retrospective study on 81 ATTR-CM patients (54 with ATTRwt) showed that diflunisal treatment for a median of one year is associated with reductions in cardiac troponin, brain natriuretic peptide (BNP), and favourable differences in echocardiographic parameters [52]. Chemical-structure modification deprived diflunisal of its cyclooxygenase inhibitory action while maintaining its TTR stabilizing effect. The new similarly structured compound was later called tafamidis [53,54].

#### 4.1.3. Tafamidis

Tafamidis meglumine (20 mg) was approved in 2011 by the EMA for stage I ATTRv polyneuropathy following the results of an 18-month phase III clinical trial (NCT00409175) where it was able to stop the disease progression in 60% of the treated compared to 38% of the placebo group [55,56]. The results of several open-label studies on patients with cardiac and neurological ATTR phenotypes warranted the investigation of tafamidis meglumine in ATTR-CM patients [57,58,59]. In 2018, the ATTR-ACT (NCT01994889) multicenter, international, double-blind, placebo-controlled, phase 3 trial, randomly assigned 441 patients with transthyretin amyloid cardiomyopathy (76% ATTRwt) in a 2:1:2 ratio to receive a daily dose of 80 mg of tafamidis meglumine, 20 mg of tafamidis meglumine, or placebo for 30 months [24]. In the composite endpoint based on a hierarchical analysis of all-cause mortality and the frequency of cardiovascular (CV) hospitalization, Tafamidis meglumine showed superiority versus placebo with a win ratio of 1.7 in a pooled approach of both doses at 18 months. Tafamidis meglumine was associated with 30% lower all-cause mortality than placebo (29.5% vs. 42.9%) and a lower rate of cardiovascular-related hospitalizations with a risk reduction of 32%. As secondary outcome parameters, tafamidis was associated with a lower rate of decline in the 6 min walk test distance and a lower rate of decline in the quality of life assessed by the score on the Kansas City Cardiomyopathy Questionnaire. Subgroup analysis showed the significant superiority of tafamidis meglumine over placebo except in patients with New York Heart Association (NYHA) class III at baseline, with an even more pronounced CV-hospitalization rate. Tafamidis meglumine was well tolerated with minor side effects, principally urinary tract infection. There was no meaningful difference in the safety of the two doses of tafamidis meglumine [24]. Higher tafamidis plasma concentrations, with doses > 60 mg tafamidis meglumine are required to achieve maximum TTR kinetic stability [60]. The results of the clinical trial supported the FDA and EMA ATTR-CM following bioequivalence studies, to grant marketing authorization of a once-daily dose of tafamidis 61 mg free acid single capsule [61]. More recently, an open-label long-term extension of the ATTR-ACT trial showed a significantly greater survival benefit with tafamidis meglumine 80 mg (4 × 20 mg) over 20 mg [62]. Further analyses from the ATTR-ACT trial showed that severely limited 6 min walk test distance (<269 m) at baseline is associated with lower mortality, but higher hospitalization compared to placebo, which can be designated clinically as the point of therapy reconciliation in NYHA III [63]. A statistical post-hoc analysis taking deaths in NYHA III patients into account could also show a benefit in CV-hospitalization rates in the survivors [64]. Based on the primary analysis with reflections to significance, the ESC granted a 1B recommendation for tafamidis in patients with hereditary ATTRv-CM or ATTRwt-CM and NYHA class I or II symptoms to reduce symptoms, CV hospitalization, and mortality [65].

#### 4.1.4. Acoramidis

Acoramidis (AG10) is an orally active TTR stabilizer under investigation. The small molecule mimics the protective TTR mutation Thr119Met by forming hydrogen bonds with the serine residues at position 117 [66]. In human plasma samples of ATTRwt, AG10 is slightly more potent as a TTR kinetic stabilizer compared to tafamidis and tolcapone. However, a dose of 1600 mg AG10 per day is required to reach the same effect of 80 mg tafamidis meglumine [67]. In a randomized, double-blind, placebo-controlled study, 49 patients with ATTRwt or ATTRm were treated with AG10 800 mg or 400 mg or placebo for 28 days. The mean changes in the serum concentration of TTR were 50%, 36%, and –7% compared to baseline, respectively. This result indicates TTR stabilization at high AG10 doses [66]. The currently ongoing ATTRibute-CM study (NCT03860935) evaluates the efficacy of AG10 800 mg b.i.d. against placebo. The study is designed to enroll 510 patients with symptomatic ATTR-CM, with an estimated completion date of May 2023. Recently, BridgeBio Pharma announced that the 12-month topline results of the ATTRibute-CM trial did not meet its primary endpoint [68].

#### 4.1.5. Bivalent TTR Stabilizers

Mds84 is a bivalent TTR ligand that simultaneously binds both binding pockets. This property overcomes the monovalent agent’s negative cooperativity binding effect [69]. In vitro, it exhibited more potent TTR stabilization compared to monovalent agents, including tolcapone and tafamidis [69,70]. However, mds84 has not been evaluated in clinical trials.

### 4.2. Transthyretin Gene Silencers

Replacing the source of aberrant TTR protein via liver transplantation has been the only effective treatment strategy for decades. ATTRv patients could only benefit from liver transplantation reaching a one-year survival rate of 74.3% and three- and five-year survival rates of 60.0% and 52.5%, respectively [71]. A paradigm-changing strategy is knocking down the gene responsible for TTR production in the liver. This is possible via small interfering RNA (siRNA) and antisense oligonucleotides (ASOs) targeting the TTR messenger RNA (mRNA) leading to its degradation before translation [72]. Knocking down the TTR gene suppresses the circulating TTR protein and disables retinol (vitamin A) transport around the body, mandating oral substitution [73]. In addition, siRNA and ASO therapies are associated with infusion-related reactions, requiring premedications or thrombopenia [71,73].

#### 4.2.1. Patisiran

Patisiran is a siRNA encapsulated in lipid-based nanoparticles enabling hepatic uptake via micropinocytosis [74]. It is the first-ever FDA-approved RNA-interfering therapy and it is indicated for Coutinho stages I and II of hereditary ATTR-polyneuropathy. Following the results of the APOLLO study, the EMA approved patisiran for mild and moderate stages of the disease. The study enrolled 225 patients with ATTRv-polyneuropathy in a placebo-controlled design. Patients who received patisiran (0.3 mg/kg every two weeks for 18 months) showed improved neurological manifestation compared to those who received placebo [75]. Typical side effects included peripheral edema and infusion reactions, mandating pre-medication with dexamethasone and histamine receptor blocker. In a subpopulation of the APOLLO study comprised of 126 patients with cardiac involvement (56% of the total population, but defined only with LV-wall thickness >13 mm in the absence of hypertension or aortic valve disease), patisiran reduced the LV wall thickness by 0.9 ± 0.4 mm, decreased global longitudinal strain by −1.4 ± 0.6%, and increased cardiac output by 0.38 ± 0.19 L/min. However, in the main cohort, there were seven deaths, all cardiovascular related, five of which were due to sudden cardiac death. In contrast, there were six deaths in the placebo group, of which only three were cardiovascular-related. It has not been elucidated whether patisiran has a causal relationship with cardiac death [76]. Recently, a small cohort of 16 hereditary ATTR-CM patients on patisiran underwent serial monitoring using cardiac magnetic resonance, echocardiography, cardiac biomarkers, bone scintigraphy, and 6-min walk tests over 12 months. Twelve patients were on concomitant diflunisal 250 mg twice daily. The results were compared to 16 retrospectively matched ATTR-CM patients who did not receive patisiran. The median serum TTR gene knockdown among treated patients was 86%. Interestingly, 82% of patients demonstrated >80% gene knockdown. Moreover, the treatment was associated with a −6.2% reduction in cardiac extracellular volume (ECV), a fall in N-terminal pro-BNP (NT-proBNP) concentrations by 1342 ng/L, an increase in 6-min walk distances by 169 m, and a median reduction in cardiac uptake by bone scintigraphy of 19.6% [77]. The APOLLO-B study (NCT03997383) is a phase III, multicenter, randomized, placebo-controlled study that investigates patisiran in ATTR-CM patients. The primary outcome measure is only the change from baseline at month 12 in the 6-min walk distance. The estimated study completion date is June 2025.

#### 4.2.2. Inotersen

Inotersen is a subcutaneously administered ASO, approved by the FDA and EMA for hereditary ATTR-polyneuropathy following the results of the NEURO-ATTR study. The approval of inotersen came two months after that of patisiran. The NEURO-ATTR study enrolled 172 patients who were randomized in a 2:1 ratio to inotrasine (300 mg every week) or placebo. Compared to placebo, the treatment group showed improved neurological manifestation, improved quality of life, and reduced mean serum TTR level by 75% from baseline [78]. Recently, Dasgupta et al. showed that long-term treatment with inotersen is safe and effective in inhibiting progression and potentially reversing the cardiac amyloid burden. Sixteen patients who completed two years of inotersen treatment showed a mean LV mass reduction of 8.4% as measured by magnetic resonance imaging, and a mean 6-min walk distance improvement by 20.2 m [79]. Today, inotersen is indicated for patients with primary hereditary ATTR-polyneuropathy stage (I–II), after the exclusion of significant ATTR-CM.

#### 4.2.3. Revusiran

Revusiran is the first N-acetylgalactosamine (GalNAc) conjugated siRNA to enter clinical trials. GalNAc is a galactose derivative that binds to the asialoglycoprotein receptor (ASGPR) on hepatocytes leading to clathrin-mediated uptake. This advancement allows dose-reduction and enables subcutaneous administration [80]. The Phase 3 ENDEAVOUR study evaluated revusiran in patients with ATTR-CM [81]. The patients were randomized 2:1 to receive subcutaneous daily revusiran 500 mg (*n* = 140) or placebo (*n* = 66) for five days over a week followed by weekly doses. The study was prematurely terminated after 6.71 months due to high mortality in the revusiran-treated arm (18 deaths) compared to the placebo-treated arm (two deaths). The subsequent analysis did not reveal any causality [81].

#### 4.2.4. Second Generation TTR Gene Silencers

Molecular modifications known as enhanced stabilization chemistry (ESC) patterns characterize second-generation RNA interference agents. ESC remarkably enhances the pharmacodynamic and pharmacokinetic properties of the RNA interference agents, allowing a significantly lower dose, with a markedly reduced dosing frequency compared to the first-generation agents [82].

Vutrisiran, formerly known as ALN-TTRsc02, is a long-acting, subcutaneously administered successor of patisiran. In a phase I, randomized, single-blind, placebo-controlled trial on 80 healthy subjects, vutrisiran demonstrated a favourable safety profile and sustained TTR plasma level reduction in a dose-dependent manner [83]. The drug is currently in a patisiran-controlled phase III trial for patients with hereditary ATTR- polyneuropathy (HELIOS-A, NCT03759379), and in a placebo-controlled phase III trial for patients with hereditary and wild-type ATTR-CM (HELIOS-B, NCT04153149). The phase III trials are estimated to be completed by May 2024 and June 2025, respectively. HELIOS-B is designed to evaluate the efficacy of vutisiran in the composite endpoint of all-cause Mortality and recurrent cardiovascular events at month 30. The study patients are 1:1 randomized to receive either vutrisiran 25 mg or placebo, subcutaneously once every three months. Recently, the drug developer announced that HELIOS-A has met all secondary endpoints measured at 18 months. In addition to improvements in exploratory cardiac endpoints including NT-proBNP, echocardiographic and scintigraphy parameters relative to placebo [84]. The FDA is currently evaluating the new drug application of vutisiran and has set an action date of April 2022.

Eplontersen, formerly known as AKCEA-TTR-LRx or IONIS-TTR-LRx, is the GalNAc-conjugated successor of inotersen. The global phase III NEURO-TTRansform study (NCT04136184) is currently evaluating eplontersen subcutaneous injections 45 mg every four weeks against inotersen 284 mg weekly in patients with hereditary ATTR-polyneuropathy [85]. In parallel, the CARDIO-TTRransform phase III study (NCT04136171) is evaluating eplontersen in ATTR-CM patients in a double-blind, randomized, placebo-controlled design. The primary endpoint is the composite cardiovascular mortality and cardiovascular event rate at week 120. The study completion date is estimated to be around June 2024.

### 4.3. Gene Editing

The recently introduced clustered regularly interspaced short palindromic repeats and associated Cas9 endonuclease (CRISPR-Cas9) gene-editing system has revolutionized biomedical research via allowing one-time treatment for deadly diseases including ATTR-CM [86]. NTLA-2001 is the first CRISPR-Cas9 therapy investigated in humans. Preclinical studies showed durable knockout of TTR after a single dose of NTLA-2001. Preliminary results from six hereditary ATTR-polyneuropathy patients enrolled in phase 1, open-label, multicenter study warrant further evaluation (NCT04601051) [87]. Three patients received a dose of 0.1 mg per kg and another three received 0.3 mg per kg. On day 28, NTLA-2001 was associated with mean TTR reductions of 52% in the first group and 87% in the second group, respectively. Mild adverse events were observed in three of the six patients [87]. The phase I study estimated completion date is August 2024.

### 4.4. Enhancing Amyloid Clearance

#### 4.4.1. Doxycycline and Tauroursodeoxycholic Acid

Doxycycline is an FDA-approved broad-spectrum antibiotic that belongs to the tetracyclines family. Tetracyclines can bind to the ribosomal subunits disrupting the synthesis of aggregation-prone proteins [88,89]. Preclinical studies on familial amyloidotic polyneuropathy mice showed that a combination of doxycycline and tauroursodeoxycholic acid (TUDCA) could remove TTR amyloid deposits and lower associated tissue markers [90]. Clinical studies have conflicting results considering the tolerability of the combination therapy. A phase II study concluded that the doxycycline/TUDCA combination is effective to halt the progression of ATTR neuropathy and cardiomyopathy with an acceptable toxicity profile [91]. In contrast, another phase II study with 28 ATTR-CM patients could only retain 36% of the enrolled patients due to adverse events including sun hypersensitivity and gastrointestinal side effects, in addition to heart function deterioration indicated by NT-proBNP elevation in the evaluated patients [92].

#### 4.4.2. Human Monoclonal Antibodies

Targeting pathological TTR amyloid deposits while sparing physiological TTR tetramers is conceivable via antibodies designed to recognize prefibrillar and fibrillar TTR-specific epitopes [93]. Antibody binding mediates the elimination of ATTR aggregates via phagocytes, which potentially leads to amyloid clearance [94]. Two drugs in this category have reached the clinical development pipeline, both designed to inhibit fibril formation via specifically targeting misfolded TTR, namely PRX004 and NI006. A phase I trial evaluating PRX004 in ATTRm patients has been recently terminated because of the COVID pandemic (NCT03336580). NI006 is currently under evaluation in a phase I, randomized, placebo-controlled, double-blind, dose-escalation trial, followed by an open-label extension phase in subjects with ATTR-CM (NCT04360434). The study completion date is estimated to be June 2022.

## 5. Discussion

**Figure 2:**
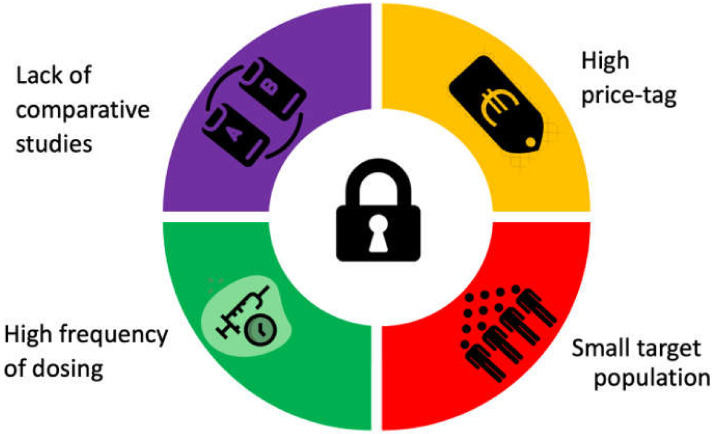
Challenges facing the clinical development of therapies for transthyretin amyloid cardiomyopathy.

ATTR-CM is an orphan disease characterized by a small patient population. The clinical development of therapies for ATTR-CM faces several challenges, as shown in Figure 2. Tafamidis (20 mg), patisiran, and inotersen are approved by the EMA (Patisiran and Inotersen by FDA also) for ATTR-polyneuropathy. Currently, only tafamidis (61 mg) is approved for ATTRv/wt-CM worldwide and recommended by the European society of cardiology guidelines as a class 1b indication in ATTR-CM patients with NYHA functional class I-II [65]. Up to now, no head-to-head comparison has been made between any of them. The three agents come at very high price tags exceeding €10,000 per month [95]. This can be attributed to the high research and development cost, the advanced technology involved in manufacturing, and most decisively the small market size. To generate sufficient revenue, orphan-designated therapeutics are high-priced [96]. The EMA offers 10 years of market exclusivity as an incentive to develop orphan-designated products. Tafamidis was granted orphan market exclusivity by the EMA based on designation EU/3/06/401. The initial price of tafamidis greatly exceeded the conventional cost-effectiveness thresholds in the US [97]. However, some price reductions are expected after the orphan market exclusivity and introduction of other stabilizers. The second wave of price reduction with generic drugs is not expected before 2026. Clinical trials in orphan diseases like ATTR-CM are challenging, the small target population results in limited power to detect the difference and greater outcome variability [98]. In part, developed treatments target the liver, being the main source of TTR. However, around 5–10% of TTR is produced in the choroid plexus and retina. A drug needs to be able to cross the blood–brain barrier (BBB) to target more neurological forms like leptomeningeal ATTR. Of the three approved medications, only tafamidis can cross the BBB. However, no more than 1.5% of the plasma-circulating drug reach the cerebrospinal fluid [99,100], which is a target of further research. Such patients may benefit from the off-label use of tolcapone. However, the use of tolcapone is limited by its hepatotoxicity and short half-life. Improving the kinetic properties of drug molecules to reduce the dose and/or the frequency of dosing has been achievable in some cases. The free acid tafamidis formulation of 61 mg allows a lower tablet consumption compared to the conventional tafamidis meglumine (4 × 20 mg). Efforts to increase the stability of the currently approved siRNA and ASOs would allow long-term TTR knock-down and less frequent dosing. Conformation-specific monoclonal antibodies are very promising and would allow clearance of the amyloid deposits without affecting the physiological TTR. Ongoing studies will determine whether ATTR-CM patients can benefit from hereditary ATTR-polyneuropathy targeting treatments. We suggest that future ATTR-polyneuropathy studies would need to include more cardiovascular-related inclusion criteria and endpoints. Direct comparative studies between different TTR-targeting treatments have been hampered by the sample size, different clinical endpoints, costs, and other reasons [101]. Post-marketing studies would be beneficial to compare the effectiveness of the different agents in the real world and to determine their superiority over each other’s taking the cost into account. In conclusion, tafamidis is the current standard of care for ATTR-CM with NYHA I-II. Patisiran and inotersen are currently used for hereditary ATTR-polyneuropathy on the way to being approved for ATTR-CM. Patients with contraindications to tafamidis or who have no insurance coverage may benefit from green tea or from the off-label use of diflunisal or tolcapone.

## Figures and Tables

**Table 1:** The use of standard heart failure therapies in transthyretin amyloid cardiomyopathy. * *except for* pulse control in uncontrollable tachyarrhythmia absoluta.

	*Yes*	*Sometimes*	*No*
Diuretics ± aldosterone antagonists	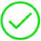		
Renin-angiotensin system inhibitors		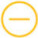	
Beta-adrenoreceptor blockers		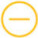	
Alpha-1-adrenoreceptor agonists		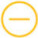	
Calcium channel blockers			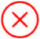
Digoxin *			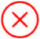

## Data Availability

Not applicable.
